# The Educational Effects of a Pregnancy Simulation in Medical/Nursing Students and Professionals

**DOI:** 10.1186/s12909-019-1589-8

**Published:** 2019-05-24

**Authors:** Jeeyoon Yu, Yoohyun Chung, Jung Eum Lee, Dae Hun Suh, Jeong Ha Wie, Hyun Sun Ko, In Yang Park, Jong Chul Shin

**Affiliations:** 1 College of Medicine, The Catholic University of Korea, Seoul, Republic of Korea; 2 Department of Obstetrics and Gynecology, Seoul St. Mary’s Hospital, College of Medicine, The Catholic University of Korea, 222, Banpo-daero, Seocho-gu, Seoul, 06591 Republic of Korea; 3 Department of dermatology, Seoul National University College of Medicine, Seoul, Korea

**Keywords:** Pregnancy, Simulation, empathy, attitude, awareness

## Abstract

**Background:**

The objective of this study was to investigate whether a pregnancy experience program (PREP) simulating physical changes in a mother during the last trimester of pregnancy could increase empathy, understanding, and positive attitude of medical/nursing students and their professional counterparts.

**Methods:**

This was a prospective observational study on medical/nursing students and their professional counterparts. Jefferson Scale of Physician Empathy (JSPE), physical difficulty and positive attitude score, and perceived effectiveness scores prior to PREP were compared to those after PREP.

**Results:**

A total of 189 participants completed PREP and questionnaires both prior to and after PREP. Mean JSPE score, physical difficulty score, and positive attitude scores were significantly increased following PREP (*p*<0.001, all). Perceived effectiveness scores about awareness, empathy, and understanding after PREP were significantly high in the professional group than in the student group (*p*=0.004, *p*=0.01, and *p*=0.017, respectively). Multiple stepwise linear regression analysis revealed that major in medicine (*p* = 0.014), health care professionals (nurse or physician) (*p*<0.001), and marriage experience (*p* < 0.001) were significant predictors of increasing empathy, difficulty feelings and effectiveness scores, respectively.

**Conclusion:**

PREP is an effective simulation program that can improve empathy, positive attitude, and awareness in medical/nursing students as well as their professional counterparts.

## Background

In 2017, the total fertility rate of South Korea dropped to 1.05 births per woman, the lowest ever recorded in the country and the lowest among OECD member countries [1]. The number of live births plummeted from 715,020 in 1995 to 438,420 in 2015 and 357,700 in 2017. The failure of various government policies to encourage childbirth is likely to stem from many factors [2, 3], such as the increased participation of women in the workforce (with no corresponding decrease in household and babysitting responsibilities), the unstable financial and employment conditions of young people, the increasing costs for having and raising a child, and insufficient affordable, safe, and hygienic public childcare facilities with reliable staff. Moreover, rapid economic growth in Korea gave rise to an extremely competitive work environment, which contributed to the current lack of positive attitudes and courtesy towards employees who require special considerations, such as pregnant women. Unfortunately, the work culture in most hospitals is not much different. Therefore, it is not easy for medical professionals to keep their empathy to patients, pregnant patients, or pregnant co-workers. However, empathy and emotional support from physicians may be particularly important for pregnant patients, because changes in hormone and neurotransmitter levels during pregnancy can cause unstable moods.

Empathy is becoming widely accepted as a prerequisite trait for current and future medical professionals, because empathy is important not only for the physician-patient relationship, but also for the treatment outcomes [4–6]. There is no one specific model for teaching empathy to medical students and physicians. However, physicians' personal experiences with illness or learning about experiences of illness through patient histories, novels, fictional stories, role playing, conversations and paintings are suggested as effective ways of teaching empathy [7]. In addition, simulation showed more effectiveness in empathy training of medical student, compared to history-based education [8]. Simulations of real-life clinical experiences conducted in a safe environment can also serve as educational deep learning approaches that enable students and professionals alike to engage in learning and make links between what they know and reality [5].

The purpose of this study was to examine whether a participation in a pregnancy experience program (PREP) that simulates the physical changes that occur in a mother during the last trimester of pregnancy increases empathy, understanding, and positive attitude towards pregnant mothers and their fetuses in medical and nursing students and in their professional counterparts.

## Methods

### Program Design

This study adopted a before and after repeated measures design. For the study, the PREP was developed with the aim of enabling participants to gain a realistic understanding of the challenges imposed by the gravid state, and also to enhance individual levels of empathy. The PREP consisted of two parts: During the first part, each participant was required to read an informational booklet that outlined the physiologic changes that occur in the maternal body during pregnancy, including weight gain, metabolic modifications, endocrine alterations, and changes in various organ systems. Next, the participant was fitted with an “empathy belly,” a pregnancy simulator in the form of an adjustable vest that allows non-pregnant wearers to temporarily experience some of the physical symptoms of the third trimester of pregnancy. A total of four empathy bellies were used for this study, all purchased from a Korean medical device company (Medicare Pharm, Bucheon, Gyeonggi Province, Korea). Each empathy belly weighed approximately 8-10 kilograms (17.6 - 22 pounds), and was equipped with artificial breasts and a protruding abdominal belly that included a battery-operated unit for the simulation of fetal kicking and the fetal heartbeat. While wearing the pregnancy simulators, participants completed simple, everyday activities, such as changing clothes, walking up and down a flight of stairs, picking up an object off the floor, and taking off and putting on their shoes. It took around 20 minutes on average for a participant to complete PREP.

### Participants

From January 21 to March 30, 2018, a total of 100 students and 100 healthcare professionals took part in the study. Participating students were currently enrolled at either the medical school or nursing school of a single university located in Seoul, South Korea. Participating healthcare professionals (HCPs) were either doctors or nurses presently working at a single university hospital, also located in Seoul. The number of participants were calculated from estimated volunteer rates as 15 percent among 860 students in the single university and the same number of HCPs in a hospital, with a 20% of drop out rate. The responses of six students and five HCPs were excluded from the final analysis due to incomplete surveys, resulting in a final total of 189 participants (94 students and 95 HCPs).

### Procedures

Prior to participation, students and medical professionals were invited to participate on a voluntary basis. All participants were asked to fill out a pre-PREP questionnaire before starting PREP and a post-PREP questionnaire just after the completion of PREP. They were provided with an explanatory statement and informed that their participation was voluntary, as well as the fact that consent was implied by their completion and submission of both the pre-PREP questionnaire and the post-PREP questionnaire. We obtained approval from the Seoul St. Mary’s Hospital institutional review boards (IRB) of the Catholic University of Korea (KC17QESI0799) and informed consent was waived by IRB.

Participants were offered a gift card with a net worth of 10,000 Korean won (about 9.30 USD) for participation. No incentives were provided to the university where the students were enrolled or to the hospital where the medical professionals were employed.

### Instruments Used

Program outcomes were measured using three instruments.

The first instrument was the Jefferson Scale of Physician Empathy (JSPE), a standardized self-reporting scale that measures empathetic attitudes in physicians and other health professionals. In this study, Korean translations of the Jefferson Scale of Empathy (JSE) version for students (S-version, JSPE-S) [9] and the JSE version for health professional (HP-version, JSPE-HP) [10] were presented to the respective participants. The validity, reliability, and internal consistency (as measured by Cronbach’s coefficient alpha) of both Korean translations has been supported by empirical studies [9].

The JSPE was originally developed to measure empathy among medical students and physicians [11]. It is a brief self-report scale with 20 items, each answered on a seven-point Likert-type format (1 = Strongly disagree, 7 = Strongly agree). Ten of the items are positively scored, the other 10 items are reverse-scored. The scores obtained for each question are then added together, resulting in a final score for empathy, which can range from a minimum of 20 to a maximum of 140. Higher scores indicate a more empathetic orientation. The scale is untimed and takes approximately 10 minutes to complete.

The second instrument was a survey developed by the researcher to measure participants’ perceptions of pregnancy and pregnant women (Survey 1) as well as attitudes towards them (Survey 2). Survey 1 consisted of questions meant to assess participants’ preconceptions of pregnancy. First, participants were asked, “Assuming you are pregnant, rate the difficulty level of the following activities: 1) Picking up an object, 2) Climbing stairs, 3) Taking off and putting on shoes, 4) Changing into and out of a t-shirt, and 5) Changing into and out of a pair of pants,” then they were asked to estimate, on a scale of 1 (extremely easy) to 10 (extremely difficult), the difficulty of performing each of the five activities while pregnant. The estimated difficulty score for each activity was termed the “Physical Difficulty Score.”

Survey 2 included thirteen five-point Likert scale questions to measure participants’ attitudes towards various aspects of pregnancy, with scores ranging from 1 (strongly disagree) to 5 (strongly agree). The score for severn item was termed the “Positive Attitude Score.”

The third instrument was a post-PREP survey (Survey 3) developed by the researcher to assess participants’ perceptions of the effectiveness of PREP. It included three 10-point Likert scale questions, with scores ranging from 1 (strongly disagree) to 10 (strongly agree) for each question. The sum total of the awareness, subjective empathy, and understanding scores was considered to be the “Perceived Effectiveness Score.”

A short demographic questionnaire was included in the pre-PREP assessment prior to the start of Section 1. The seven demographic questions assessed participants’ occupation and level (either in school or in the hospital), gender, and age. The participants were also asked “Do you have family or friends who are pregnant,” “Have you lived or interacted with a pregnant woman in a communal setting,” “Have you ever been pregnant,” and “Have you ever been married.”

#### Statistical Analysis

Statistical calculations were performed with SPSS (version 24.0, Chicago, IL, USA), including means and proportions. Chi square tests were performed to compare proportions of independent variables while t-tests were performed to compare means. A paired repeated measures t-test was used to compare before and after results. Stepwise multiple linear regression analysis was performed in order to find significant predictors of the change of JSPE and physical difficulty scores, before and after PREP, and perceived effectiveness score after PREP. Statistical significance was considered to have been reached with a P value of <0.05.

## Results

### Participant Demographic Characteristics

A total of 189 participants completed PREP and the before and after questionnaires, with a preponderance of women overall (67.2%), within the student cohort (53.2%), and within the professional cohort (81.1%) (Table 1). Most participants were in the 21-30 age group (65.6%), followed by the 31-40 age group (19.6%), 41-50 age group (11.6%), and 10-20 age group (3.2%). The majority of participants did not have family or friends who were pregnant at the time (79.9%). There were more participants with no experience living or interacting with pregnant women (70.9%) than those who did have such experience (29.1%). A more detailed description of the participants is given in Table 1.

**Table 1:** Participant Demographic Characteristics

	Total (*n* =189)		Students (*n* = 94)		Professionals (*n* = 95)		
Category	No.	(%)	No.	(%)	No.	(%)	*P*
Gender							<0.001
Male	62	(32.8)	44	(46.8)	18	(18.9)	
Female	127	(67.2)	50	(53.2)	77	(81.1)	
Major							<0.001
Medical Students and Doctors	112	(59.3)	74	(78.7)	38	(40)	
Nursing Students and Nurses	77	(40.7)	20	(21.3)	57	(60)	
Age Group							<0.001
10-20	6	(3.2)	6	(6.4)	0	(0)	
21-30	124	(65.6)	88	(93.6)	36	(37.9)	
31-40	37	(19.6)	0	(0)	37	(38.9)	
41-50	22	(11.6)	0	(0)	22	(23.2)	
Do you have family or friends who are pregnant?							<0.001
Yes	38	(20.1)	4	(4.3)	34	(35.8)	
No	151	(79.9)	90	(95.7)	61	(64.2)	
Have you lived or interacted with a pregnant woman in a communal setting?							<0.001
Yes	55	(29.1)	15	(16)	40	(42.1)	
No	134	(70.9)	79	(84)	55	(57.9)	
Have you ever been pregnant?							<0.001
Yes	29	(15.3)	0	(0)	29	(30.5)	
No	160	(84.7)	94	(100)	66	(69.5)	
Have you ever been married?							<0.001
Yes	35	(18.5)	0	(0)	35	(36.8)	
No	154	(81.5)	94	(100)	60	(63.2)	

Data are presented as number (%). Independent t-tests were performed between students and healthcare professional groups

### JSPE Scores

Cronbach’s alpha was 0.85 for the pre-PREP JSPE and post-PREP JSPE, indicating very high internal consistency. The mean JSPE score in the whole group was 103.71±13.52 prior to PREP, and this increased significantly to 107.04±14.39 after PREP (p<0.001) (Table 2). The mean JSPE score of participants majoring in medicine increased significantly after PREP (103.51±13.82 vs 106.81±14.65, p<0.001), and that of participants majoring in nursing also increased significantly after the PREP as well (104.01±13.16 vs 107.38±14.08, p<0.001). In terms of difference of JSPE scores, there was significantly higher change in the medical students/doctors than nursing students/nurses (*p* < 0.001), between two groups. There was significant improvement of JSPE score within male and female group, as well as within student and professional group (*p* < 0.001, all). However, there was no significant difference of score change, between the groups. There were significant improvements of JSPE score in 21-30 yeas old and 31-40 years old groups, but not in 11-20 years old and 41-50 years old groups. However, there was no significant difference of score change, among the age groups.

**Table 2:** Jefferson Scale of Physician Empathy (JSPE) Scores and difference of JSPE scores, Before and After the Pregnancy Experience Program (PREP)

	JSPE score before-PREP	JSPE score after-PREP	*P* ^*^	Difference of JSPE scores before and after PREP	*P* ^**^
Total Participants (*n* = 189)	103.71±13.52	107.04±14.39	<0.001	3.33±7.41	
Gender					0.164
Male (*n* = 62)	104.73±12.93	107.06±13.86	<0.001	4.4±7.74	
Female (*n* = 127)	103.56±13.85	106.79±14.69	<0.001	2.8±7.21	
Major					<0.001
Medical Students or Doctors (*n* = 112)	103.51±13.82	106.81±14.65	<0.001	4.42±7.36	
Nursing Students or Nurses (*n* = 77)	104.01±13.16	107.38±14.08	<0.001	1.74±7.23	
Age group (years old)					0.438
11-20 (*n* = 5)	103.8±12.74	106.8±12.64	0.142	3±3.67	
21-30 (*n* = 125)	104.77±14.12	107.92±14.95	<0.001	3.61±7.8	
31-40 (*n* = 37)	101.19±11.32	105.73±12.48	<0.001	3.86±7.42	
41-50 (*n* = 22)	101.95±13.69	104.32±14.86	0.172	0.91±5.28	
Status					0.432
Students (*n* = 94)	107.7±12.54	111.46±12.89	<0.001	3.76±7.72	
Professional (*n* = 95)	99.77±13.35	102.67±14.52	<0.001	2.91±7.1	

Data are presented as mean±standard deviation. *JSPE* Jefferson Scale of Physician Empathy, *PREP* Pregnancy Experience Program, *n* number. *paired t-test within a group, **t-test between groups and ANOVA test among 4 groups

### Physical Difficulty Scores

Cronbach’s alpha was 0.91 for the pre-PREP physical difficulty score and 0.88 for the post-PREP physical difficulty score, indicating very high internal consistency. The mean total physical difficulty score in the whole group increased significantly from 36.12±7.67 before PREP to 39.95±7.62 after PREP (*p* < 0.001) (Table 3).

**Table 3:** Comparisons of Physical Difficulty Scores Before and After the Pregnancy Experience Program (PREP)

		Physical Difficulty Scores (0-10)		
Physical Activities		Before PREP	After PREP	P*
Picking up Object		7.34±1.64	7.7±2.01	0.027
Climbing Stairs		7.41±1.69	7.79±1.87	0.019
Putting on and Taking off Shoes		7.23±1.89	8.03±1.79	<0.001
Changing T-Shirt		6.81±1.98	8.35±1.87	<0.001
Changing Pants		7.33±1.76	8.08±1.77	<0.001
Total		36.12±7.67	39.95±7.62	<0.001

Data are presented as mean±standard deviation. PREP: Pregnancy Experience Program. *paired t-test

### Positive Attitude Scores

Cronbach’s alpha was 0.7 for the pre- PREP positive attitude score and 0.72 for the post- PREP positive attitude score, indicating very high internal consistency. Table 4 shows the changes in the positive attitude scores of the participants from pre-PREP assessment to post-PREP assessment. The positive attitude scores in all seven items were significantly changed after PREP (*p* < 0.001, all)

**Table 4:** Positive Attitude Scores Before and After Pregnancy Experience Program (PREP)

	Positive Attitude Scores (0-5)		
	Pre-PREP	Post-PREP	p*
When I meet a pregnant woman, I feel a special sympathy towards her.	2.82	3.42	<0.001
When pregnant, even doing everyday tasks will be difficult.	3.76	4.26	<0.001
Pregnant women should receive special consideration for the fact that they are pregnant.	4.14	4.36	<0.001
I would understand a pregnant woman's difficulties and give her special consideration.	4.08	4.35	<0.001
If I (or my girlfriend) were pregnant, my way of life would change significantly.	4.09	4.32	<0.001
Feeling fetal movement will increase my respect for life.	4.16	4.37	<0.001
Feeling fetal movement will significantly limit my physical activity.	3.84	4.25	<0.001

*JSPE* Jefferson Scale of Physician Empathy, *PREP* Pregnancy Experience Program

*Paired t-test

### Elements of the Perceived Effectiveness Score

**Fig. 1:**
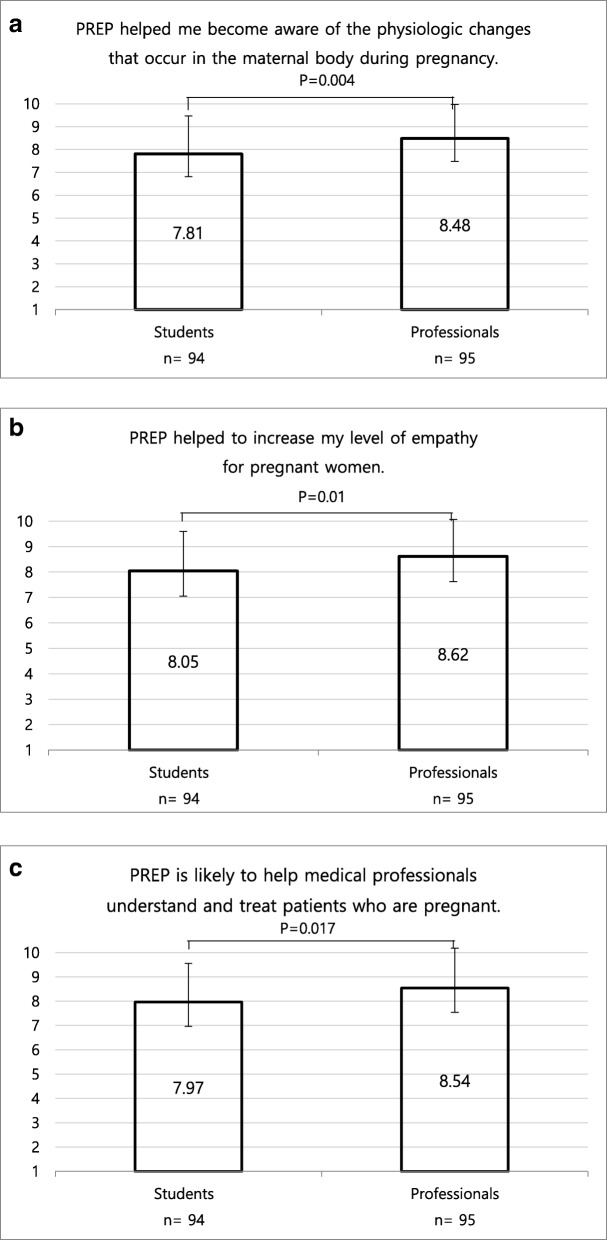
Perceived Effectiveness Scores After the Pregnancy Experience Program (PREP). **a** Awareness, **b** Subjective empathy, **c** Understanding scores were compared between students and professionals groups

Cronbach’s alpha was 0.95 for the perceived effectiveness score, indicating very high internal consistency. The perceived effectiveness score was assessed only after PREP and the scores were compared between the professional cohort and student cohort. The awareness score associated with participants’ awareness of the physiologic changes that occur in the maternal body during pregnancy (Figure 1) was significantly higher in the professional cohort than in the student cohort (8.48±1.49 vs 7.81±1.65, *p* = 0.004). The subjective empathy score associated with increased empathy towards pregnant women was also significantly higher in the professional cohort than in the student cohort (8.62±1.45 vs 8.05±1.55, *p* = 0.01). Lastly, the understanding score associated with increased understanding of pregnant patients was also found to be significantly higher in the professional cohort than in the student cohort (8.54±1.65 vs 7.97±1.59, *p* = 0.017).

### Multiple Linear Regression Analysis

Regarding the JSPE score change before and after PREP, the results of the multiple linear regression analysis showed that only ‘major’ of participants was a significant predictor (Table 5). Majoring in medicine was a significant predictor of a greater change in empathy level after PREP, as compared to majoring in nursing (*p* = 0.014). Regarding the total physical difficulty score change, only status (i.e. student or professional) was confirmed to be a significant predictor. Being a professional was a significant predictor of physical difficulty score change, as compared to students (p < 0.001). Regarding the perceived effectiveness score, marriage experience was confirmed to be the only significant predictor for high effectiveness score. Participants who had marriage experience had significantly higher perceived effectiveness scores than those with no experience of marriage (*p* < 0.001).

**Table 5:** Significant Predictors in change of Jefferson Scale of Physician Empathy and Physical Difficulty score, before and after PREP, and in Effectiveness Scores after PREP by Stepwise Multiple Linear Regression Analysis

Factors	Factors Affecting JSPE Score change			Factors Affecting Total Physical Difficulty Score change			Factors Affecting Perceived Effectiveness Score		
	Estimate	95% CI	*P*	Estimate	95% CI	*P*	Estimate	95% CI	*P*
Gender									
Female	Ref			Ref			Ref		
Male	0.004		0.966	-0.03		0.672	0.03		0.687
Major									
Nursing	Ref			Ref			Ref		
Medicine	2.679	(0.545, 4.813)	0.014	0.047		0.524	0.01		0.905
Age									
Every 10-year increase	-0.009		0.914	0.024		0.782	0.077		0.431
Status									
Student	Ref			Ref			Ref		
Professional	0.015		0.848	6.245	(3.916, 8.574)	<0.001	0.098		0.223
Have experience living with a pregnant woman									
No	Ref			Ref			Ref		
Yes	-0.033		0.659	-0.05		0.487	0.1		0.223
Have experienced pregnancy									
No	Ref			Ref			Ref		
Yes	0.028		0.733	0		0.996	-0.202		0.198
Have experienced marriage									
No	Ref			Ref			Ref		
Yes	0.071		0.381	0.069		0.371	3.053	(1.43, 4.677)	<0.001

Null hypothesis: No difference in scores by factor. *PREP* Pregnancy Experience Program, *JSPE* Jefferson Scale of Physician Empathy, *CI* Confidence Interval

## Discussion

The main objectives of this study were to: (1) assess the levels of empathy, understanding, and positive attitude of aspiring and current health professionals towards pregnant women and fetuses prior to participating in a pregnancy simulation program, and (2) compare the pre-PREP findings with post-PREP results. We observed a significant increase in self-assessed empathy levels by JSPE in all cohorts after participation in PREP. There have been many studies utilizing JSPE score to assess empathy levels in healthcare students or professionals, although these have found mixed results. Systematic reviews have found that physician empathy appears to be an important aspect of patient and physician well-being, and targeted educational programs may significantly enhance the empathy levels of medical students and physicians [11–15]. However, other studies have demonstrated that brief interventions did not lead to significant increases in empathy [16, 17]. These mixed conclusions may be due to the limitations of self-assessment, as one randomized controlled trial found that participants in the intervention group showed significantly higher levels of empathy than the control group when rated by simulated patients and experts, but no significant group differences were observed in self-rated empathy [8].

We found that the only significant predictor of change of empathy level (as indicated by JSPE scores) after PREP was the major (medicine or nursing) of the participants. A previous study found that doctors and nurses had similar levels of empathy [6]. The other study demonstrated that medical students showed lower empathy level than nursing students [18]. In this study, the absolute empathy level towards pregnant women was higher in nursing students or nurses than in medical students or doctors. However, this study also shown that PREP was more effective in increasing empathy towards pregnant women among medical students or doctors than among nursing students or nurses.

In this study, gender was found to be not a significant factor of change of JSPE score after PREP. This is inconsistent with previous literature indicating that females show greater levels of empathy than males in general [19–21]. However, there have also been several reported studies in which no gender difference was observed [22–24]. This study suggests that PREP is an effective intervention in increasing empathy, regardless of gender.

In addition, our findings also suggest that understanding of pregnant women and their fetuses increased after participation in PREP, as indicated by the significant increases of physical difficulty score in the whole group. The only significant predictor of physical difficulty score change was the status (student or professional) of the participants. Being a professional was a significant predictor of high physical difficulty score change, as compared to students (*p* < 0.001). It seems that students are physically strong and empathy belly can make less burden to them, because all participants of students were less than 30 years old and there were significantly higher percentage of males in student group than in professional group.

Lastly, the perceived effectiveness score, an exclusively post-PREP self-assessment measuring awareness, subjective empathy, and understanding of pregnancy, was significantly higher in the professional cohort than in the student cohort, indicating that PREP has a greater effect on participants who are already working in the field. The only significant predictor of high perceived effectiveness score was marriage experience.

There are several limitations to this study. One limitation of this study is that the convenience sample may not be representative of all students or medical professionals due to the lack of randomization of participants. It was also conducted at a single institution, restricting the generalizability of the results. Further, our study did not have another groups trained by using a different teaching method, such as classical lecture or self-reading. The observed increase in the post-PREP figures may also have been overestimated due to the Hawthorne effect (the tendency for people to perform better when they are participants in an experiment and are being observed) as well as the desire of health professional students and health professionals to be portrayed in a positive light. These phenomena may have influenced their responses on the self-reporting instrument, irrespective of their experiences. In addition, evaluations immediately following educational interventions can overestimate the effects of these interventions [25]. Moreover, mean difference of JSPE score was 3.33 (from 103.71±13.52 to 107.04±14.39, *p* < 0.001), before and after PREP, in this study. It can be considered as a small change after an intervention. However, various modules in increasing empathy need to be developed and validated to maintain empathy in students and HCPs [26]. This was a pilot study with small sample size and limit conclusions about generalizability of findings. More research evaluating the effectiveness of PREP and the maintenance of increased empathy levels over longer periods and on a larger scale is needed to confirm our findings. To the best of our knowledge, this was the first study to simultaneously evaluate empathy levels and attitudes regarding pregnancy among healthcare students or HCPs. Although there have been studies that have employed pregnancy simulation [27] or an infant simulator [28] to educate adolescent students about pregnancy, the main purpose of these interventions was to reduce the risk of unintentional adolescent pregnancy in the participants rather than to increase their empathy level.

Simulation is a tool increasingly used in education, and it is considered as an appropriate educational method for teaching empathy to healthcare students [29]. This is the first study that demonstrates the effectiveness of a pregnancy simulation program in increasing objective empathy in healthcare students and professionals.

## Conclusion

Our findings suggest that pregnancy simulation programs may increase empathy, understanding, and positive attitude towards pregnant women and their fetuses in current and future healthcare professionals. More larger scaled studies need to be followed to confirm this finding, investigate maintenance duration of the effect of PREP and investigate other useful educational programs to keep or promote increased empathy in healthcare students and professionals.

## Abbreviations

HCP: Healthcare Professional
JSPE: the Jefferson Scale of Physician Empathy
JSPE-HP: JSE version for health professional
JSPE-S: the Jefferson Scale of Empathy (JSE) version for students
OECD: Organization for Economic Cooperation and Development
PREP: Pregnancy Experience Program
